# Supplementary material for: Relationship between Microflora Changes and Mammary Lipid Metabolism in Dairy Cows with Mastitis
Source: Animals (Basel). 2023 Aug 31;13(17):2773. doi: 10.3390/ani13172773 (PMC10486416; doi:10.3390/ani13172773)
Supplement: Supplementary file 1 [file animals-13-02773-s001.zip › animals-2506768-supplementary.pdf]

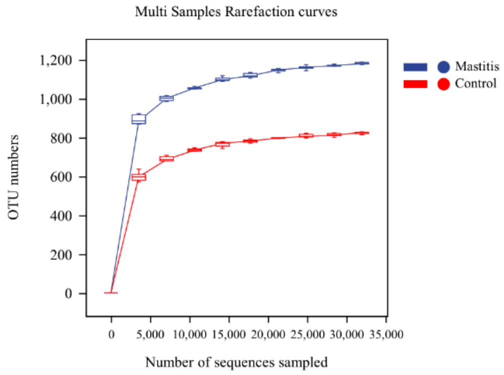

Figure S1 Rarefaction curves for milk samples. The abscissa is the drawing depth, and the ordinate is the median value and boxplot of alpha diversity index calculated for 10 times.

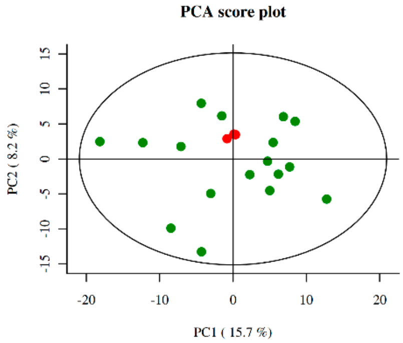

Figure S2. PCA score of QC samples. The abscissa PC1 and the ordinate PC2 represent the scores of the first and second principal components, respectively. Each scatter represents a sample, and the color and shape of the scatter represent different groups. The sample is basically in the 95% confidence interval.

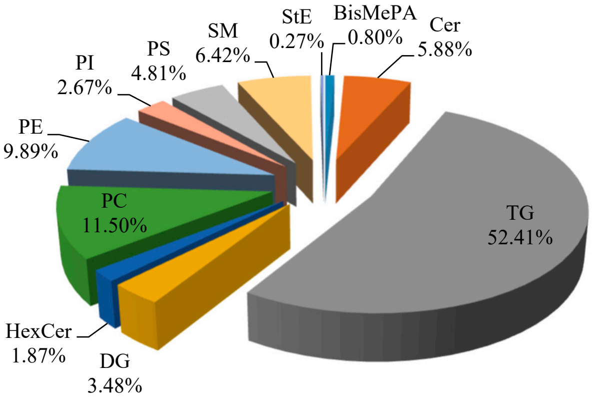

Figure S3. Lipid classes identified. Each sector in the pie chart of lipidomics results represents a class of metabolites, and its size represents its percentage. PI, phosphatidyl inositol; PE, phosphatidyl ethanolamine; PS, phosphatidylserine; SM, sphingomyelin; DG, diglyceride; TG, triglyceride; StE, stigmasteryl ester; Cer, ceramides; HexCer, hexaglycosylceramide; BisMePA, Bis methylphosphatidic acid.
